# The Use of Zebrafish (*Danio rerio*) Behavioral Responses in Identifying Sublethal Exposures to Deltamethrin

**DOI:** 10.3390/ijerph110403650

**Published:** 2014-04-02

**Authors:** Yi Huang, Jinsong Zhang, Xiaobo Han, Tinglin Huang

**Affiliations:** 1 School of Environmental and Municipal Engineering, Xi’an University of Architecture and Technology, Xi’an 710043, China; E-Mails: huangyiwater@163.com (Y.H.); huangtinglin@xauat.edu.cn (T.H.); 2 Shenzhen Water (Group) Co., Ltd., Shenzhen 518031, China; E-Mail: xiaobohan7@gmail.com; 3 Research Center for Eco-Environmental Sciences, Beijing 10085, China

**Keywords:** behavioral response, bio-monitoring, zebrafish, hyperactivity, surfacing

## Abstract

Alterations of fish behavioral responses are sensitive indicators to identify accidental chemical pollution. In this research, a series of exposure tests were conducted to investigate behavioral changes of adult zebrafish (*Danio rerio*) exposed to deltamethrin (DM) in six concentrations of 0, 0.15, 1.5, 3.75, 7.5 and 15 μg/L. Swimming changes in zebrafish were detected at a concentration as low as 1% of the LC_50-24h_ within five hours. Hyperactivity was the first response, followed by a second response of fish surfacing. The change patterns of swimming speed in zebrafish were similar in all exposure groups, but the degree increased with increasing concentrations. Swimming speed and depth were altered within the first two hours after exposure, which was regarded as the most vital phase for water quality monitoring. The duration of hyperactivity and the time of zebrafish surfacing were both logarithmically correlated with exposure concentrations, which was helpful to distinguish the level of pollution.

## 1. Introduction

Pyrethroid insecticides are being used as viable substitutes for organochlorine and organophosphate ones in pest-control programs because of their low environmental persistence and toxicity. The use of pyrethroids as insecticidal and antiparasitic formulations has markedly increased and recently accounted for over 30% of all insecticides used globally [1]. Deltamethrin (DM) is one of the most popular synthetic pesticides because of its limited pesticide persistence in soil, high activity against a broad spectrum of insect pests and low toxicity to non-target organisms [2]. The widespread use of DM has consequently led to high concentrations in surface waters and it has been found to be highly toxic to fish, mussels, some beneficial aquatic arthropods and finally, to possibly cause toxic effects to the public [3]. Recent studies have found potential hazards of deltamethrin to fetuses, infants, and children [4,5]. There is therefore a great and urgent demand to rapidly detect accidental or deliberate contamination by deltamethrin, due to its potentially severe consequences to the environment and human health.

Fish are one of the most important aquatic organisms because of their economic value and their sensitivity to contaminants, and have been used in a wide range of biological assays. Compared with *Daphnia magna*, shellfish and other aquatic organisms, fish can last for more than one month without feeding and are more suitable for long-term monitoring of accidental discharges [6]. The rapid behavioral responses seen in fish make them ideal subjects for observation, and analysis of fish behavior has been a popular approach to detect changes in the aquatic environment [7]. Viran *et al.* [8] found the responses of loss of equilibrium and hanging vertically in the water of guppy fish (*Poecilia reticulata*) when exposed to DM at all concentrations above 4 μg/L. Floyd *et al.* [9] also demonstrated that fish experiencing acute exposures to sublethal concentrations of pyrethroid insecticides exhibited significant behavioral impairment. The effects of DM on fish biomarkers have been extensively investigated and reviewed, but studies on fish behavior for water monitoring are still insufficient [10].

In China, deltamethrin is a commonly used pesticide for pest control in the agricultural fields around freshwater reservoirs and is one of the most important risk materials causing sudden and accidental pollution. Zebrafish have been widely used as a prominent model organism in monitoring and assessing the effects of contaminants in the aquatic eco-environment, both because they provide invaluable comparative material for work on mammals and humans, and because it’s easy to determine their numbers and behaviors, they are inexpensive, low-maintenance, and produce abundant offspring. In the current study, a typical sequence of fish behavior toxicity tests were conducted to measure the effects of different concentrations of DM on zebrafish in the form of LC_50_ values, swimming speed and depth. Understanding the behavioral change patterns of zebrafish under sudden stress caused by chemicals assists in the development and application of water quality criteria for the protection of the aquatic environment.

## 2. Experimental Section

### 2.1. Test Species and Test Chemical

Zebrafish were obtained from the Institute of Hydrobiology and kept under a constant photoperiod of 10:14 (L:D) and fed three times per day. Dechlorinated water with a dissolved oxygen concentration of 6.8 ± 0.2 mg/L was used and the temperature was maintained at 28.5 ± 1 °C. Adult zebrafish of similar lengths (30 ± 2 mm), age (6–8 months) and body weight (300 ± 2 mg) were selected and acclimated for three weeks in a glass tank before experiments. Deltamethrin, ((*S*)-α-cyano-3-phenoxybenzyl (1*R*,3*R*)-3-(2,2-dibromovinyl)-2,2-dimethylcyclopropan-1-carboxylate, CAS Registry No.: 52918-63-5), was obtained from the Shanghai Pesticides Research Institute (Shanghai, China).

### 2.2. Experimental Setup

#### 2.2.1. Acute Toxicity Test

Experiments were performed according to the Organization for Economic Cooperation and Development (OECD) standard method [11] to determine the LC_50-24h_ of zebrafish. Mortality was controlled 6, 12 and 24 h after the start of the tests. Dead individuals were removed immediately. Acute toxicity test was carried out according to Polat *et al.* [12].

#### 2.2.2. Behavioral Response Monitoring

**Figure 1:**
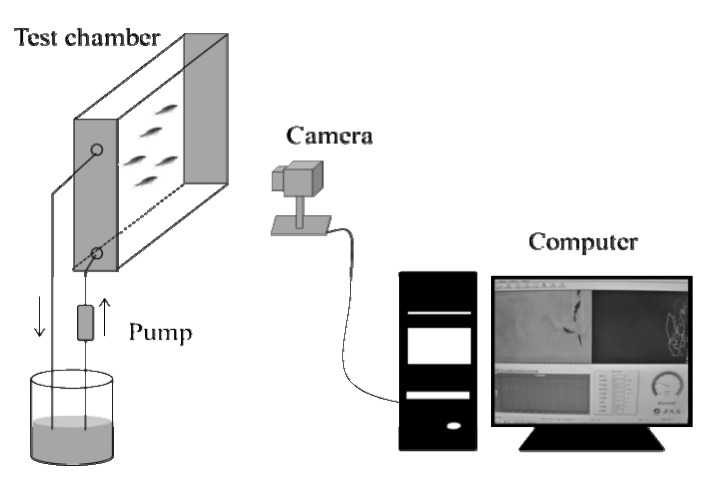
Experimental setup for behavioral measurement of zebrafish.

Subsequently, six groups of fish (*n* = 5) was exposed to 0, 0.15, 1.5, 3.75, 7.5 and 15 μg/L of DM for behavior monitoring. Measurements were performed at the same period of the day, under similar illuminance level (200 lx) and without noise. Three male and two female adult zebrafish were selected in each experiment and then transferred into the rectangular test tank (400 × 75 × 300 mm). A real-time CCD camera (SDZ-371P; Samsung, Tianjin, China) was placed in the front of the test tank to record the swimming trajectories of the five zebrafish (Figure 1).

Images from the camera were sent to a computer at nearly 10–12 frames per second and then converted into two-dimensional data, *x* and *y* coordinates, which facilitated subsequent quantification of fish behavior including average swimming speed and depth. In order to reduce the signal capture errors, the median of speed and depth at 1 min intervals were extracted for analysis in this study. The test solution was delivered by a pump (AP3100, JEBO, Zhongshan, China) with a continuous cycle flow through system (flow rate, 1 L/min). Experiments were performed according to Barry [13] and Beauvais *et al.* [14]. The swimming behavior of the test fish was recorded and observed for a total of 6 h. In the first hour, dechlorinated water was delivered for recording the unexposed behavior as a reference for the toxicity assessment. Then, the test solution was released into the test chamber for 5 h.

### 2.3. Data Analysis

We performed statistical analysis of fish responses under exposed and unexposed conditions at 1 min intervals for behavioral parameters. The values during each interval were compared to the values during the initial 60 min of the exposure test before exposure (unexposed conditions). Differences in endpoints were checked for assumptions of homogeneity of variance across treatments by Levene’s test, analysed by one-way analysis of variance (ANOVA) and then tested by Dunett’s test. When homogeneity was not observed, nonparametric statistical comparisons (Wilcoxon-test) were used to detect differences between unexposed and exposed conditions. Statistical tests were done using the SPSS 13.0 computer program (SPSS Inc. Chicago, IL, USA). Differences between means were considered significant when *p* < 0.05.

## 3. Results

### 3.1. Acute Toxicity Test

The results obtained from acute static 24 h toxicity experiments of DM for zebrafish and estimated LC_50_ values, slope and confidence limits are listed in Table 1. The LC_50-24h_ of DM for zebrafish was found to be 14.43 ± 1.031 μg/L. Rapid gill movement, erratic swimming, swimming in a corkscrew manner, rapid opercular movement, swimming at the water surface and “gulping for air” were observed clearly in all concentrations above 7 μg/L. Fish were less active or inactive, remaining vertically in the water or laid on one side and in some cases still at the bottom of the tank just moments before death. Previous studies indicated the high toxicity of DM to fish species and our results were in good agreement with previous reports [8,15].

**Table 1:** Acute toxicity (LC_50-24h_), slope and 95% confidence limits of DM to zebrafish.

Pesticide	Slope	LC_50-24h_ (μg/L)	95% Confidence Limit	
			Upper limit	Lower limit
Deltamethrin	5.4621	14.43 ± 1.031	15.36	13.56

### 3.2. Behavioral Responses of Zebrafish to DM

**Figure 2:**
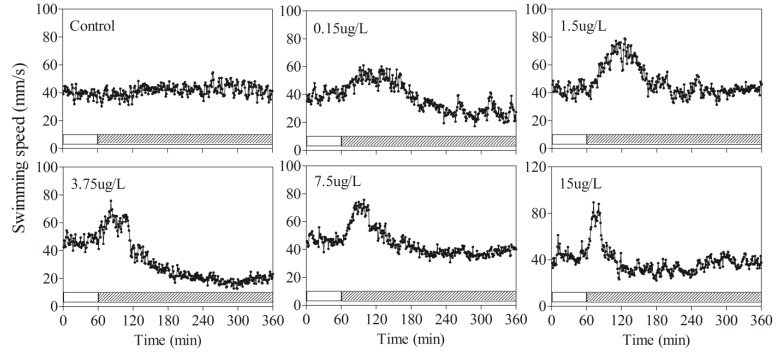
The effects of sublethal DM concentrations on the behavioral changes of zebrafish. The solid and hatched bars at the bottom of graphs indicated the period in which unexposed and exposed swimming speed were measured, respectively.

Although variations existed between individuals, the data obtained from our behavioral monitoring system showed that fish behavior in the control group remained stable over the experimental period. The behavior did not vary significantly between the control group and unexposed condition in the toxic exposure tests (Table 2, *p* > 0.05). The speed of zebrafish varied mainly in a certain range of 25–60 mm/s (Figure 2).

**Table 2:** Summary of statistical comparisons of fish responses under unexposed conditions and under exposure to DM. Data represent Mean ± SD of three independent experiments. The values during each 1-min interval were compared to the interval of the initial 60 min.

Time Interval (min)			Concentration (μg/L)					
			0	0.15	1.5	3.75	7.5	15
**Speed**	**Average**	0–60	39.6 ± 5.8	39.6 ± 1.1	42.3 ± 2.5	48.7 ± 3.4	46.7 ± 3.8	43 ± 4.4
		60–120	38 ± 4.7	49.7 ± 5.9	58.6 ± 6.7	58 ± 9.9	61.2 ± 7.8 *	54 ± 6.8 *
		120–180	42.6 ± 4.3	48.6 ± 1.5	57.6 ± 9	32.3 ± 9.5	46.7 ± 11.2	31.3 ± 9.6
		180–240	42.3 ± 3.5	32.8 ± 1.9	42.3 ± 9.2	22 ± 5.4 *	38.4 ± 3.2	31.1 ± 11.8
		240–300	44 ± 6.7	27.0 ± 5.8	41.9 ± 18	18.2 ± 16.9 *	37.3 ± 9.5	36.9 ± 12
		300–360	41.8 ± 10.5	28.06 ± 7.18	42.5 ± 12.9	19.4 ± 16 *	38.6 ± 7.8	38.2 ± 14.1
	**Max**	0–60	52 ± 7.3	55.7 ± 4.3	57.4 ± 4.8	61.3 ± 1.4	59.9 ± 11.2	60.5 ± 0.6
		60–360	60.6 ± 8.3	64.26 ± 2.8	87.1 ± 13.4 *	79.8 ± 9.3 *	84.9 ± 9.4 *	99.5 ± 5.4 *
**Depth**	**Average**	0–60	156.9 ± 17	177.1 ± 25.4	173.9 ± 28.7	157.5 ± 25.6	126.1 ± 21.2	131.5 ± 36.3
		60–120	142.1 ± 9.8	179.2 ± 11.9	187.8 ± 6.5	163.1 ± 10.2	178 ± 19.6	164.2 ± 41.1
		120–180	135.4 ± 24.2	169.5 ± 10.9	189.1 ± 31.5	193.3 ± 14.2 *	181.2 ± 36.7 *	199.4 ± 38.3 *
		120–240	128.7 ± 13.7	176.6 ± 15.2	198.6 ± 15.2	189.3 ± 8.8 *	212.9 ± 9.7 *	219.9 ± 21 *
		240–300	144.5 ± 17.5	160.2 ± 31	209.8 ± 5 *	168.7 ± 37.6	192.2 ± 27.6 *	244.7 ± 7.6 *
		300–360	137.2 ±19.9	171.7 ± 36.7	221.9 ± 3.4 *	121.9 ± 47.1	215.2 ± 31.8 *	228.8 ± 5.4 *
	**Max**	0–60	232 ± 22.8	241.5 ± 28.8	237.3 ± 23	236.2 ± 2.6	191.8 ± 21.2	216.7 ± 21.2
		60–360	252.5 ± 15.8	258.4 ± 9.1	271.8 ± 14.5	294.2 ± 10.2 *	285.2 ± 25 *	269.9 ± 6.1 *

Note: * a significant differences from control (*p* < 0.05).

**Figure 3:**
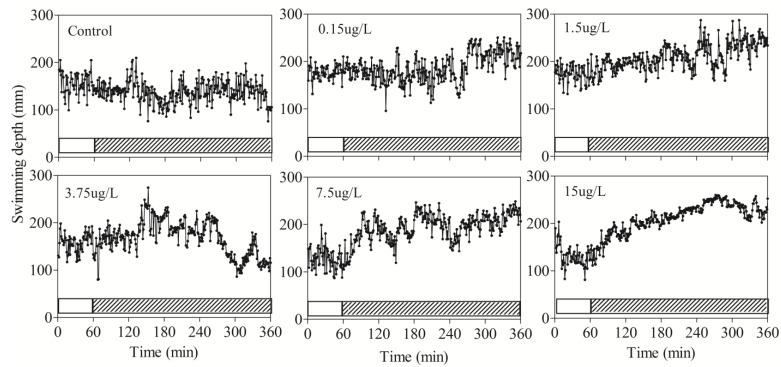
The effects of sublethal DM on swimming depth of zebrafish. The hatched and solid bars at the bottom of graphs indicated the period in which unexposed and exposed swimming speed were measured, respectively.

Zebrafish were distributed uniformly across the vertical position intervals, and the average depth was mostly within the range from the 100 to 200 mm (Figure 3). Therefore, these results were taken as standards for the entire experimentation.

The behavioral response patterns of zebrafish exposed to DM in all concentration tests were similar and time-dependent. Exposures to DM significantly increased swimming speed during the initial stages of exposure, however, after a period of hyperactivity, the decreased sharply to equal or less than unexposed conditions (Figure 2). As shown in Table 2, the swimming speed in the lowest concentration (0.15 μg/L) was increased from the average 39.6 mm/s to 49.7 mm/s, and increased in the highest DM concentration (15 μg/L) from 43.0 mm/s to 54.0 mm/s. 

**Figure 4:**
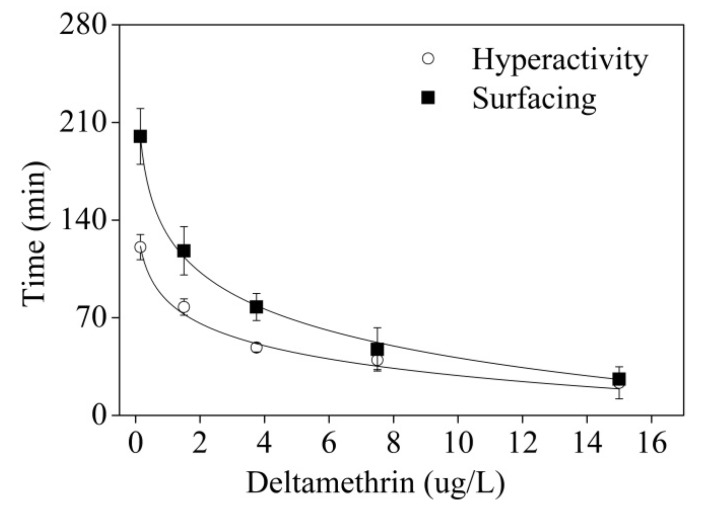
Time until detection of abnormal behaviour in a concentration-dependent manner. Reductions in the duration time of hyperactivity and the time swimming depth starting to change were significantly correlated with increased concentration (*r*^2^ = 0.9510, *p* < 0.05 and *r*^2^ = 0.9969, *p* < 0.05, respectively). All error bars represent SD of group means.

The maximum speed increased with increasing concentration (Figure 4), even up to 99.5 mm/s for 15 μg/L exposure. Higher exposure concentrations led to greater changes of swimming activity, but shorter hyperactivity duration time. After a period of hyperactivity, fish slowly became lethargic, restless, and secreted excess mucus all over the body. The average speed decreased to equal or lower than unexposed conditions.

Unlike the dramatic changes of speed, swimming depth suffered a gradual increasing trend after exposure. Zebrafish surfaced more frequently than unexposed fish and the swimming depth fluctuated within the range from 200 to 300 mm in each exposure concentration (Figure 3). However, these changes did not occur immediately after exposure. Higher concentration resulted in an earlier accumulation to reach the threshold value of hypoxia in a shorter time. The swimming depth in the lowest concentration (0.15 μg/L) was higher than 200 mm after 210 min exposure but only after 13 min exposure in the highest DM concentration (15 μg/L).

There were also obvious concentration-response relationships between DM and the behavioral responses of zebrafish. Compared to the controls, the duration time of hyperactivity of zebrafish in the lowest concentration (0.15 μg/L) DM lasted for nearly 140 min, followed by a gradually decreasing trend. At the highest DM concentration (15 μg/L), the fish were less tolerant and the behavioural strength changed very rapidly, with only 23 min from sudden increase and sharp decrease to a lower level. The mean duration of hyperactivity and exposure concentration were significantly correlated (logarithm correlation (y = 82.8 − 23.52 × ln(x − 0.04), *r*^2^ = 0.9510, Figure 4). Swimming depth increased immediately after speed recovered at the highest concentration exposure (15 μg/L), started to increase within 30 min after hyperactivity with medium concentration exposure (3.75 μg/L) and 120 min after hyperactivity for the lowest concentration exposure (1.5 μg/L). Exposure concentration and the time of swimming depth starting to change were also significantly correlated (logarithm correlation (y = 129.31 − 38.23 × ln(x − 0.006), *r*^2^ = 0.9969, Figure 4). By comparing the duration of hyperactivity and the time until detection of surfacing, it could be identified that the time gap between them decreased with the increased concentration.

## 4. Discussion and Conclusions

Despite the variations between individuals, swimming speed and depth remained stable over the experimental period under control conditions and significantly increased in the lowest concentration exposure (0.15 μg/L). Alterations in swimming behavior have been detected during exposures to various contaminants at concentrations as low as 0.7% to 5% of their LC_50_ values [16]. This study showed that swimming changes in zebrafish could be detected for concentrations of DM as low as 1% of the LC_50-24h_ within five hours. Behavioral responses are faster and more sensitive toxicity parameters than mortality, which also indicated that fish behavioral changes were more suitable for the on-line monitoring for accidental discharges of DM.

The results of this study suggested that the behavioral responses of zebrafish to different DM concentrationss tended to be similar. In all of the experimental treatments, swimming speed increased significantly during the initial stages of exposure, followed by a loss of movement and increased swimming depth. Organisms display a time-dependent sequence of behavioral stress responses during exposure to pollutants above their respective thresholds of resistance [17]. The concentrations in this study were above the threshold concentration and swimming speed increased to escape the polluted aquatic environment during the first few minutes after exposure (avoidance behavior). Higher concentrations led to greater increases in fish behavioral strength, but shorter duration time during the first phase behavioral response. The maximum increase rate was 68% in the 0.15 μg/L group and the duration of hyperactivity lasted for almost 140 min. In the 15 μg/L exposure, by contrast, the maximum was 189% and hyperactivity only sustained for 23 min. Certain DM concentrations would be too high for zebrafish to maintain high behavioral strength and then the activity of fish locomotion tended to decrease gradually until the second responses occur under continued exposure. The increase of swimming activity when aquatic animals encounter pollutants was also observed in several studies of other species such as Japanese medaka (*Oryzias latipes*), Estuarine calanoid copepod (*Eurytemora affinis*) [10], *Gammarus pulex* [18] and *Rotifera* [19].

A frequently studied physiological correlate of behavioral change is brain cholinesterase activity. Beauvais *et al.* [14] and Brewer *et al.* [20] found a correlation between changes in swimming speed and brain ChE activity induced by exposure to diazinon and malathion. Deltamethrin has also been proved to be an inhibitor or stimulator of acetylcholinesterase (AChE) activity in the brain of zebrafish causing severe neurotoxicity, which may correlate positively with changes in swimming speed [21]. Higher concentration made more obvious changes in the activity of AChE, which led to the greater degree of hyperactivity. Similar results have been observed in natural ecosystems polluted with organophosphates, carbamates, and other pollutants [22,23].

If the first response of hyperactivity resembled an escape reaction allowing zebrafish to evade stressful conditions, surfacing behavior was a result of a chemical exposure. With increasing exposure time, chemicals accumulated in tissues, such as gills, liver and kidney, and caused tissue damage. DM can be highly absorbed by the fish gills and caused severe respiratory impairment because of its lipophilic features, which made fish stay near the water surface to get more oxygen [24]. The range of swimming depth changed from 100–200 to 200–300 mm in each exposure concentration, which indicated that zebrafish preferred to stay within the area just below the water-surface. Kang *et al.* [25] also indicated that the duration of surfacing behavior was a useful endpoint to analysis the effects of KCN, NaCN, and aldicarb on Japanese medaka. However, the time until detection of staying on the surface has a 20–200 min delay compared with the hyperactivity phenomenon. Higher concentrations led to greater behavioral changes and metabolism, causing a greater accumulation to reach the threshold value of hypoxia in a shorter time [26], so the time gap between hyperactivity and surfacing decreased more, especially in the 7.5 and 15 μg/L groups. Gerhardt [27] also proved that the ventilation damage resulted from a higher threshold of both response intensity and contaminant concentration.

The results showed that the first two hours after exposure to DM were the most important phase for monitoring water quality by fish behavioural changes. As the first response to chemicals, avoidance with increased speed was considered as the most important endpoint. However, hyperactivity also presented by other physiological stimuli, such as sudden noise, strong light and other disturbances, which were the primary cause of false alarms of these fish behavioral monitoring systems in water quality applications [28]. A second response of changes in swimming depth later on with continued exposure, might be a good standard to discern between true and false system warnings. The duration of hyperactivity and the time of zebrafish starting to change were both logarithmically correlated with exposure concentration, which was helpful to distinguish the level of pollution.

In addition, there were only two endpoints used in this paper (swimming speed and depth), so further research on the complexity of the trajectory and characteristic of fish schools is required to increase the significance and usefulness of fish behavioral indicators for water quality monitoring or ecotoxicological effect assessment.
